# Effect of Human Milk Appetite Hormones, Macronutrients, and Infant Characteristics on Gastric Emptying and Breastfeeding Patterns of Term Fully Breastfed Infants

**DOI:** 10.3390/nu9010015

**Published:** 2016-12-28

**Authors:** Zoya Gridneva, Sambavi Kugananthan, Anna R. Hepworth, Wan J. Tie, Ching T. Lai, Leigh C. Ward, Peter E. Hartmann, Donna T. Geddes

**Affiliations:** 1 School of Chemistry and Biochemistry, The University of Western Australia, Crawley, Perth, Western Australia 6009, Australia; 21141062@student.uwa.edu.au (S.K.); anna.hepworth@uwa.edu.au (A.R.H.); ash.tie@uwa.edu.au (W.J.T.); ching-tat.lai@uwa.edu.au (C.T.L.); peter.hartmann@uwa.edu.au (P.E.H.); donna.geddes@uwa.edu.au (D.T.G.); 2 School of Anatomy, Physiology and Human Biology, The University of Western Australia, Crawley, Perth, Western Australia 6009, Australia; 3 School of Chemistry and Molecular Biosciences, The University of Queensland, St. Lucia, Brisbane, Queensland 4072, Australia; l.ward@uq.edu.au

**Keywords:** human milk, term breastfed infants, gastric emptying, feeding frequency, ultrasound, stomach volumes, appetite hormones, macronutrients, feed volume, anthropometrics, body composition

## Abstract

Human milk (HM) components influence infant feeding patterns and nutrient intake, yet it is unclear how they influence gastric emptying (GE), a key component of appetite regulation. This study analyzed GE of a single breastfeed, HM appetite hormones/macronutrients and demographics/anthropometrics/body composition of term fully breastfed infants (*n* = 41, 2 and/or 5 mo). Stomach volumes (SV) were calculated from pre-/post-feed ultrasound scans, then repeatedly until the next feed. Feed volume (FV) was measured by the test-weigh method. HM samples were analyzed for adiponectin, leptin, fat, lactose, total carbohydrate, lysozyme, and total/whey/casein protein. Linear regression/mixed effect models were used to determine associations between GE/feed variables and HM components/infant anthropometrics/adiposity. Higher FVs were associated with faster (−0.07 [−0.10, −0.03], *p* < 0.001) GE rate, higher post-feed SVs (0.82 [0.53, 1.12], *p* < 0.001), and longer GE times (0.24 [0.03, 0.46], *p* = 0.033). Higher whey protein concentration was associated with higher post-feed SVs (4.99 [0.84, 9.13], *p* = 0.023). Longer GE time was associated with higher adiponectin concentration (2.29 [0.92, 3.66], *p* = 0.002) and dose (0.02 [0.01, 0.03], *p* = 0.005), and lower casein:whey ratio (−65.89 [−107.13, −2.66], *p* = 0.003). FV and HM composition influence GE and breastfeeding patterns in term breastfed infants.

## 1. Introduction

Breastfeeding and its longer duration are associated with reduced risks of developing obesity and other chronic non-communicable diseases later in life [1,2]. This unique protection could be the result of many mechanisms associated with both nutritive and non-nutritive components of human milk (HM) [3] as well as breastfeeding patterns and behaviour [4,5]. It has been shown that HM has the pleiotropic role, providing immune and anti-inflammatory protection [6,7] and endocrine, developmental, neural, and psychological benefits [2]. Non-nutritive HM components such as hormones, growth factors, neuropeptides, and anti-inflammatory and immune-modulating agents influence the growth, development, and function of the gastrointestinal (GI) tract during early infancy [8], while some micronutrients act as nutritional antioxidants, improving GI functions [9]; however, there is much to be learned about the spectrum of HM programming agents, how their patterns change throughout lactation period, and their short-term effect on the gastric emptying (GE) rate of the breastfed infants.

GE is a process by which ingested food is mechanically and chemically partially broken down and delivered to the duodenum at a controlled rate for further digestion and absorption [10,11]. While well studied in the preterm population [12,13,14], in healthy term fully breastfed infants the GE rate and its relationship with breastfeeding patterns are not fully understood.

GE rate and patterns are known to depend on the nature and macronutrient composition of the ingested meal. HM or formula in the infant stomach separates into two phases, a liquid phase consisting of water, whey proteins, lactose, etc., and a semi-solid phase consisting of curd formed by casein and lipids. The semi-solid phase typically empties more slowly than the liquid phase. Different proportions of these phases in part explain the difference between GE patterns of formula-fed and breastfed infants—linear and curvilinear, respectively [12,15].

HM has a unique composition, including nutrients, growth factors, immune factors, and hormones. Despite numerous investigations into the different effects of HM and formula, few components, including major macronutrients, have been studied in connection with the GE of breasted term infants.

Fatty acids profiles are not associated with GE rate in preterm infants [16], while in term infants more rapid GE has been attributed to the fat and protein components of feeds with similar lactose concentration and osmolality [17].

Both osmolality and carbohydrate content are known to influence the rate of GE in adults [18], but in infants results are dependent on the type of carbohydrate [19,20].

Proteins from different HM fractions such as whey and casein are resistant to proteolysis in the infant stomach [21] and the protein content of a food has also been shown to influence appetite and its regulation [22]. Infant formula generally empties more slowly than HM in term infants; further, formulas with different casein:whey protein ratio exhibit different GE rates, with casein-predominant formulas emptying slower than whey-predominant formulas [23]. Thus the casein:whey ratio of HM could play an important role in controlling GE in the breastfed infant.

HM lysozyme, also present in whey in a relatively high concentration, catalyzes the hydrolysis of specific bonds in Gram-negative bacteria cell walls and plays multiple roles in digestive strategy, such as controlling the microbiome in the stomach and speeding up the digestion of microbial protein, which may affect gastric motility and GE rate [24,25].

The satiety hormone leptin and the appetite-stimulating hormone adiponectin are also present in HM. Although not transferred to the infant circulation in direct manner, levels of HM leptin and adiponectin from HM have been found to correlate with levels of these hormones in infant serum [26,27] and are known to affect both appetite control and infant body composition (BC) [28,29], but are yet to be investigated in relation to GE in the term infant. In animal models (rat, mouse), injection of leptin into the fourth ventricle has been shown to delay GE [30] and oral administration reduced food intake [31]. Leptin in HM is by far the most studied appetite hormone, but predominantly in skim milk [32]. Leptin measured in skim HM was not associated with time between feeds [33,34] or GE [34] in term breastfed infants, emphasizing the need for studies including whole milk leptin, where the levels of leptin are shown to be higher [32]. Adiponectin has the highest concentration of any appetite hormone in HM. It is present in a biologically active form that is resistant to digestion [35]. In the animal model adiponectin inhibits tension-sensitive gastric vagal afferent mechanosensitivity, modulating satiety signals in both lean and obese animals, while simultaneously increasing the mechanosensitivity of mucosal gastric vagal afferent in the obesity-induced model [36]. In humans, elevated serum levels of adiponectin are associated with more rapid GE in diabetic patients [37]. It is not known whether adiponectin levels impact GE in the infant and this warrants further investigation.

The volume of milk taken at a single feed varies greatly both within and between infants [38]. This may be affected by HM composition, with greater breastfeeding frequency associated with lower total 24-h protein intakes and higher lactose concentrations [39]. This suggests that the variations in HM components between mothers may potentially influence GE rate and time, and therefore feeding patterns.

This study investigated the effects of HM appetite hormones (whole milk adiponectin and leptin, skim milk leptin) and macronutrients (fat, total carbohydrates, lactose, oligosaccharides, total protein, casein and whey protein, lysozyme) on feeding frequency and GE. Further exploration of infant demographics, anthropometrics, and BC was carried out to determine relationships with infant feeding and GE.

## 2. Materials and Methods

### 2.1. Participants

Lactating mothers and their infants (*n* = 27) were recruited predominantly through the Australian Breastfeeding Association. Inclusion criteria were: healthy singletons, gestational age ≥ 37 weeks, fully breastfed on demand at the point of measurement. Exclusion criteria were: infant health issues requiring medication that could potentially influence GE rate (e.g., reflux), indications of low maternal milk production or infant growth issues. All mothers provided written informed consent to participate in the study, which was approved by the University of Western Australia, Human Research Ethics Committee (RA/1/4253) and registered with the Australian New Zealand Clinical Trials Registry (ACTRN12616000368437).

### 2.2. Study Design

Participants arrived at our laboratory at King Edward Memorial Hospital for Women (Subiaco, Perth, WA, Australia) in the morning (09:30–11:30 a.m.) to avoid circadian influence on the outcomes, and stayed for two consecutive breastfeeding sessions. Before the first feed (F1) infants were weighed and had ultrasound stomach volumes recorded (pre-feed residual, R1). Mothers expressed a pre-feed sample (fore-milk) of milk from the feeding breast/breasts and then breastfed their infants as usual. Immediately after F1, infant stomach volumes images and infant weights were taken, and mothers expressed a post-feed (hind-milk) milk sample. Subsequent scans of the stomach were scheduled at 15–20 min intervals (although attending infants’ needs caused some variation) until the infant cued for the next feed (F2), when a final stomach volume immediately before F2 was measured (pre-feed residual, R2).

To assess infant BC bioimpedance spectroscopy measurements were taken pre-feed, unless impractical—then they were taken post-feed [40]. Ultrasound skinfold, length, and head circumference measurements were taken post-feed. This combination of two methods for measuring infant BC was used to ensure safe, non-invasive and accurate assessment and to avoid the inherent limitations of a singular technique [41]. Clothing was removed for the measurements except for a dry diaper and a singlet.

### 2.3. Feeding Frequency

Mothers were asked how frequently their infant feeds, and the self-reported typical time between the feeds (e.g., every three hours) during the week prior to the study session was taken as a proxy measure of feeding frequency.

### 2.4. Feed Volume Measurement

The volume of milk transferred from a breast/breasts by the infant was determined by weighing the infant immediately before and after the breastfeed using electronic scales (±2.0 g, Medela Electronic Baby Weigh Scales, Medela Inc., McHenry, IL, USA). Milk intake (g) was calculated by deducting the initial weight from the final weight of the infant [42] and was converted to mL (feed volume; FV) using HM density of 1.03 g/mL [43].

### 2.5. Stomach Measurements with Ultrasound

**Figure 1:**
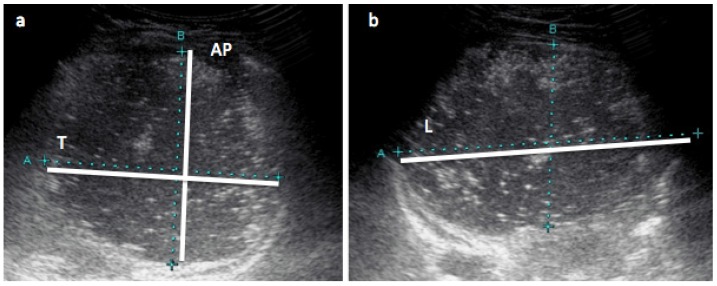
Measurements of infant’s stomach with ultrasound. Ultrasound images of infant’s stomach: (**a**) transverse view with anterior-posterior (AP) and transverse (T) diameter measurements; (**b**) longitudinal view with longitudinal (L) diameter (maximum length) measurement. Stomach volume (mL) = longitudinal diameter (mm) × anterior-posterior diameter (mm) × transverse diameter (mm) × 0.52.

The infant’s stomach was scanned using the Aplio XG (Toshiba, Tokyo, Japan) machine, with a high-resolution PVT-674BT (6MHz) transducer and Parker ultrasonic gel (Fairfield, NJ, USA). Three to nine (median [IQR]: 5 [5; 6]) serial measurements of infant stomachs were taken 3 to 62 min apart (16 ± 10). Scans were performed with the infant in the semi-supine position according to the method validated in preterm infants [44]. Briefly, the sagittal and transverse planes of the stomach were used to measure the longitudinal (L), anterior-posterior (AP) and transverse (T) diameters directly from images on the ultrasound screen using electronic calipers (Figure 1). One experienced sonographer with good intra- and interrater reliability [44] performed all of the measurements. Gastric volume (mL) was calculated from the above measured diameters using following equation for an ellipsoidal body:

*Stomach volume* (mL) = L (mm) × AP (mm) × T (mm) × 0.52. (1)

### 2.6. Milk Sample Collection

Mothers hand-expressed or pumped small (1–2 mL) pre- and post-feed milk samples into separate 5-mL polypropylene plastic vials (Disposable Products, Adelaide, SA, Australia). Fat concentration was measured (below) and samples were frozen at −20 °C for further biochemical analysis.

### 2.7. Biochemical Analysis

#### 2.7.1. Fat Content

Percentage fat was measured in pre- and post-feed samples immediately after sample collection with the creamatocrit method [45] using the Creamatocrit Plus device (Medela Inc., McHenry, IL, USA). Fat concentration of the pre- and post-feed milk samples (g/L) was calculated from the cream content of the milk samples, based on the equation [46]:

*Fat* (g/L) = 3.56 + (5.917 × cream percentage). (2)

Fat concentration in the volume consumed by the infant was further calculated [47]:

*Fat* (g/L) = 0.53 × Fat _pre-feed_ + 0.47 × Fat _post-feed_. (3)

#### 2.7.2. Sample Preparation

Prior to further analysis, all samples were thawed for two hours at room temperature (RT) and aliquoted into 1.5-mL tubes (Sarstedt, Numbrecht, Germany). Components’ concentrations were determined in both pre- and post-feed samples in case of adiponectin, skim and whole milk leptin, fat, and lactose, and in pooled samples in case of total protein, casein, whey protein, total carbohydrates, and lysozyme. Concentrations of pre- and post-feed samples were averaged to arrive at the concentration used for statistical analyses. Whole milk was used for measuring whole milk adiponectin and leptin concentration. Milk samples were defatted (by centrifugation at RT in a Beckman Microfuge 11 (Aberdon Enterprise Inc., Elk Grove Village, IL, USA) at 10,000× *g* for 10 min and removing the fat layer by clipping it off with the top of the tube [48]) for analysis of skim milk leptin, total protein, lysozyme, lactose, and total carbohydrates concentrations. The standard assays were adapted for and carried out using a JANUS workstation (PerkinElmer, Inc., Waltham, MA, USA) and measured on EnSpire (PerkinElmer, Inc., Waltham, MA, USA).

#### 2.7.3. Leptin

Leptin concentration in HM was measured using the R & D Systems Human Leptin enzyme linked immunosorbent assay (ELISA) DuoSet kit (Minneapolis, MN, USA) optimized to measure leptin in sonicated skim HM, as previously described by Cannon et al. [33] and further modified to measure leptin in skim and whole HM milk as described by Kugananthan et al. [32]. Recovery of leptin was 97.7% ± 9.7% (*n* = 10) with a detection limit of 0.05 ng/mL and an inter-assay CV of <7.2%.

#### 2.7.4. Adiponectin

Adiponectin concentration in whole milk was measured using the Biovendor Human Adiponectin Sandwich ELISA kit (Life Technologies, Asheville, NC, USA). Adiponectin recovery was 96.2% ± 3.2% (*n* = 10) with a detection limit of 1 ng/mL and an inter-assay CV of <2.5%.

#### 2.7.5. Protein

Casein and whey proteins were separated by the method fully described by Kunz and Lonnerdal [49], and Khan et al. [50]. Protein concentrations (total protein of skim HM, casein and whey proteins) were measured using the Bradford Protein Assay adapted from Mitoulas et al. [51]. Recovery of protein was 100.6% ± 5.2% (*n* = 5) with a detection limit of 0.031 g/L and an inter-assay CV of 7.8% (*n* = 18). Casein:whey ratio was calculated as follows:

*Casein:whey ratio* = casein concentration/whey protein concentration. (4)

#### 2.7.6. Lysozyme

Lysozyme concentration was determined using a modified turbidimetric assay [52]. Hen egg white lysozyme (EC 3.2.1.17, Sigma, St. Louis, MA, USA) standards (range 0.00075–0.0125 g/L) and skim milk samples were diluted 10-fold with 0.1 M of Na_2_HPO_4_/1.1 mM of citric acid (pH 5.8) buffer. Twenty-five microliters of standards or diluted skim milk samples were placed into the wells of a plate (Greiner Bio-One, Frickenhausen, Germany), 175 μL of *Micrococcus lysodeiltikus* suspension (0.075% *w*/*v*, ATCC No. 4698, Sigma, St. Louis, MA, USA) was added into each well and plate was incubated at RT for 1 h. The absorbance was measured at 450 nm. Recovery of lysozyme was 97.0% ± 5.0% (*n* = 8) with a detection limit of 0.007 g/L and an inter-assay CV of 13.0% (*n* = 8).

#### 2.7.7. Carbohydrates

Defatted milk was deproteinized with trichloroacetic acid [53] before dehydration by sulphuric acid [54]. This technique reliably estimates concentrations and carbon content for monosaccharides, disaccharides, and polysaccharides. Total carbohydrates were analyzed by UV-spectrophotometry. Recovery of total carbohydrates was 101.4% ± 2.1% (*n* = 7) with a detection limit of 0.007 g/L and an inter-assay CV of 3.3% (*n* = 7).

Lactose concentration was measured using the enzymatic spectrophotometric method of Kuhn and Lowenstein [55], adapted from Mitoulas et al. [51], with recovery of 98.2% ± 4.1% (*n* = 10), detection limit of 30 mM and inter-assay CV of 3.5%.

The human milk oligosaccharides (HMO) concentration (g) was calculated by deducting concentration of lactose (g) from concentration of total carbohydrates (g). The glucose and galactose were not measured or accounted for as their concentrations in HM are small and comparable or less than the assays errors [56].

### 2.8. Hormone and Macronutrient Dose

Doses were defined as the amount of hormone/macronutrient ingested during a breastfeed and calculated as average of the pre- and post-feed HM component concentration, multiplied by the corresponding FV. When an infant fed from both breasts at the breastfeeding session, hormone/macronutrient doses from these individual breastfeeds were calculated separately and added together.

### 2.9. Infants’ Anthropometrics and Body Composition

#### 2.9.1. Anthropometric Measurements

Infants’ weight was determined by weighing before breastfeeding using Medela Electronic Baby Weigh Scales (±2.0 g; Medela Inc., McHenry, IL, USA). Clothing was removed except for a dry diaper and a singlet. Infant crown-heel length was measured once to the nearest 0.1 cm using non-stretch tape and headpiece and footpiece, both applied perpendicular to the hard surface. Infant head circumference was measured with non-stretch tape. Infant BMI was calculated according to the following formula:

*BMI* = Body weight (kg)/(Height (m))^2^. (5)

#### 2.9.2. Body Composition with Bioelectrical Impedance Spectroscopy

Infants’ whole body bioimpedance were measured using the Impedimed SFB7 bioelectrical impedance analyzer (ImpediMed, Brisbane, Queensland, Australia) applying an adult protocol (wrist to ankle) according to the manufacturer’s instructions and analyzed with settings customized for each infant according to Lingwood et al. [57] and Gridneva et al. [41]. Values of resistance (ohm) at frequency of 50 kHz (R_50_) were determined from the curve of best fit, averaged for analysis purposes and used in the Lingwood et al. age matched (3 and 4.5 mo infants) equations for fat-free mass (FFM) of 2 and 5 mo infants respectively [57]:

*FFM 3* mo = 1.458 + 0.498 × W − 0.197 × S + 0.067 × L^2^/R_50_ (6)

*FM 4.5* mo = 2.203 + 0.334 × W − 0.361 × S + 0.185 × L^2^/R_50_, (7)

where L is body length (cm), R_50_ is resistance (Ω), S is sex (male = 1, female = 2) and W is infant weight (kg).

%FM was calculated as follows:

%FM = 100(Weight (kg) − FFM (kg))/Weight (kg).
(8)

#### 2.9.3. Body Composition with Ultrasound Skinfold Measurements

Infant ultrasound skinfold measurements were carried out using the Aplio XG (Toshiba, Tokyo, Japan) ultrasound machine, PLT-1204BX 14-8 MHz transducer and sterile water-based Parker ultrasonic gel (Fairfield, NJ, USA). Single ultrasound scans of four anatomical sites (biceps, subscapular, suprailiac, and triceps) were performed on the left side of the body with minimal compression. Skinfold thickness (skin thickness and the skin–fat interface to fat–muscle interface distance) was measured directly from images on the screen using electronic calipers. One experienced sonographer (DG) with good intra- and interrater reliability [44] performed all of the measurements.

The doubled ultrasound skinfold thickness was used in Brook body density (*d*) age-matched (3–18 mo) equations [58] developed for skinfolds measured with calipers:

*Male d* = 1.1690 − 0.0788 × log (∑SFT) (9)

*Female d* = 1.2063 − 0.0999 × log (∑SFT), (10)

where *d* is infant body density (kg/L) and ∑SFT is a sum of four skinfolds (mm).

Predicted body density was converted to %FM using the Lohman equation [59]:

*%FM* = 100 × (5.28/*d* − 4.89), (11)

where *d* is the infant body density (kg/L).

### 2.10. Statistical Analysis

Statistical analysis was performed in R 2.9.0 [60] for Mac OSX using additional packages nlme [61]; lattice [62], lattice extra [63], and car [64]; MASS [65], sfsmisc [66] and multcomp [67] for mixed effects modeling, data representation, robust regression, and multiple comparisons of means, respectively. Descriptive statistics are reported as mean ± standard deviation (SD) (range) or median (IQR) unless otherwise stated; model parameters are presented as estimate ± standard error (SE), and, where appropriate, an approximate 95% confidence interval (95% CI).

Measurements missing due to insufficient sample volume: skim milk leptin, whole milk leptin, adiponectin, total protein, whey and casein protein, lactose and total carbohydrate (*n* = 3); lysozyme (*n* = 5). Measurements of fat (*n* = 14) were missing as a result of either insufficient sample volumes or absence of separate feed volumes from breasts where both breast were offered during one feed. Also missing were feeding frequency as reported by mothers (*n* = 6), measurements of length, head circumference, infant BMI, %FM measured with bioelectrical spectroscopy (*n* = 4) and %FM measured with ultrasound skinfolds (*n* = 5).

GE time was determined as the time from the start of F1 to the start of F2 and included the time between two feeds and feed duration. Feed duration was included as up to 80% of HM consumed by term healthy breastfed infants in the first 4–5 min [68]. GE during breastfeeding was defined as the volume of milk to have left the stomach, calculated as the difference between the immediate post-feed stomach volumes and the sum of R1 and FV.

Due to the lack of term infant gastric-emptying studies focusing on stomach volume, no power calculation/sample size determination could be performed for this study. A goal of 20 infants at each two and five months was selected with the expectation that this would be sufficient to show overall patterns. When available, infants were included in both subsets to allow for investigation of longitudinal patterns. Linear mixed effects models allow us to treat the individual feeds as separate, without having to assume independence, when there may be correlations between feeds within infants.

Influences on GE rate were analyzed by first fitting a time curve to the sequential post-feed stomach volumes using linear mixed effects models; as curves differed significantly within and between infants (*p* < 0.001), random time curves were fitted to feeds within infants. Time terms (linear, square root) were selected as per the fractional polynomial method of [69]; this model also considered possible confounding effects of FV (median-centred) and feed duration (median-centred). Interaction terms involving the time curve indicated changes in the GE rate; main effects indicated overall effects on post-feed stomach volumes but not the GE rate. The addition of one term to this base model was used to investigate associations with (a) concentrations/doses of hormones/macronutrients; (b) infant characteristics/anthropometrics/BC; (c) R1. Whether the overall effect of HM component concentrations differs by feed volume was investigated by including interactions between FV and concentration measures. Models using the selected technique did not converge for fat concentration, lysozyme concentration, or lysozyme dose. Omitting the random effect of feed within infant provided converging models, but no evidence of an association with fat or lysozyme was seen. Given the complexity of linear mixed effects models used to analyze GE rate, no further adjustments were performed and *p* < 0.05 was considered to be statistically significant.

Associations between pre-feed residual stomach volumes, FV, immediate post-feed stomach volumes, feed duration, feeding frequency and both hormone and macronutrient concentrations and doses, and infant anthropometrics/BC parameters were tested using robust linear regression. Mixed effects models were considered, but were not significantly better (*p* > 0.1) Robust linear regression (rlm) was chosen so as to address heteroscedasticity in the data and points with high leverage in the majority of the predictors; MM-estimation (M-estimation with Tukey’s biweight, initialized by a specific S-estimator) accounting for appropriate covariates was used [65]. Approximate p-values were determined using the Wald test. Multivariate models accounting for FV were used for testing the relationship with FV-dependent predictor (fat dose and concentration).

Possible age differences in HM components, infant characteristics, and GE/breastfeeding parameters were analyzed with either linear mixed effects models or robust linear regression models; model type was determined using likelihood ratio tests. Linear mixed effects models were used to analyze relationships of GE during feed time with HM components and infant characteristics. R1, FV and feed duration were not associated with stomach volume reduction during the feed time, therefore univariate models were run. Multivariate linear mixed effects models accounting for R1, FV and feed duration were used in analysis of relationships of immediate post-feed stomach volumes with HM components and infant characteristics.

Owing to the large number of comparisons, a false discovery rate adjustment [70] was performed on associated subgroupings of results with one or more *p*-values < 0.05. *p*-values were considered to be significant at <0.011 for GE time, <0.031 for feeding frequency, <0.038 for R2, and <0.008 for associations between HM components’ concentrations.

## 3. Results

### 3.1. Participants

Characteristics of the 27 participants (2 months (*n* = 20; longitudinal: 7 females, 7 males; cross-sectional: 2 females, 4 males); 5 months (*n* = 21; longitudinal: 7 females, 7 males; cross-sectional: 6 females); overall *n* = 41 feeds) are described in Table 1. At the study session, infants fed from one (*n* = 23) or both (*n* = 18) breasts.

### 3.2. Influence of Infant Age

Infant anthropometrics and %FM measured with bioimpedance spectroscopy significantly differed by infant age (*p* < 0.001), while breastfeeding and GE parameters did not change significantly (*p* > 0.067) (Table 1).

Lower whey protein concentration (5.51 ± 0.96 g/L 5 mo vs. 6.41 ± 1.39 g/L 2 mo, *p* = 0.034) and subsequently a higher casein:whey ratio (0.32 ± 0.14 5 mo vs. 0.22 ± 0.07 2 mo, *p* = 0.035) were observed at 5 months. All other measured appetite hormones and macronutrient concentrations did not differ significantly by infant age (*p* > 0.053).

### 3.3. Analyzed Human Milk Components

Appetite hormones and macronutrient concentrations and doses per feed are presented in Table 2. Higher skim milk leptin concentrations were associated with lower whole milk leptin concentrations (−0.25 [−0.34, −0.16], *p* < 0.001) and higher protein concentrations were associated with higher whey protein concentrations (0.68 [0.41, 0.95], *p* < 0.001). Higher HMO concentrations were associated with higher total carbohydrates concentrations (*p* < 0.001) and lower lactose concentrations (*p* < 0.001).

### 3.4. Gastric Emptying Rate

**Figure 2:**
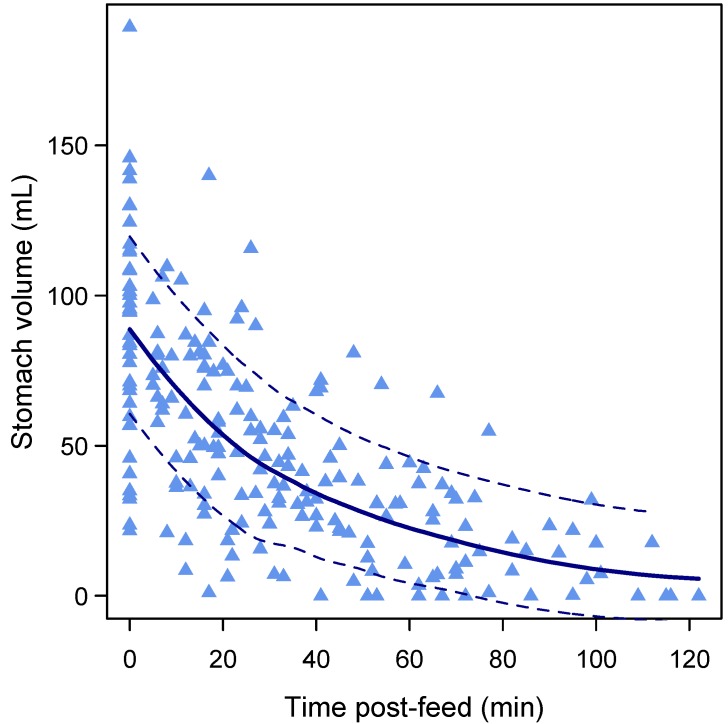
Overall curvilinear pattern of gastric emptying (*n* = 41 feeds). The lines represent the overall pattern of changes in stomach volume as measured by ultrasound imaging. Bold line represents local regression smoother (LOESS, span = 0.9). Dotted lines represent confidence interval.

**Figure 3:**
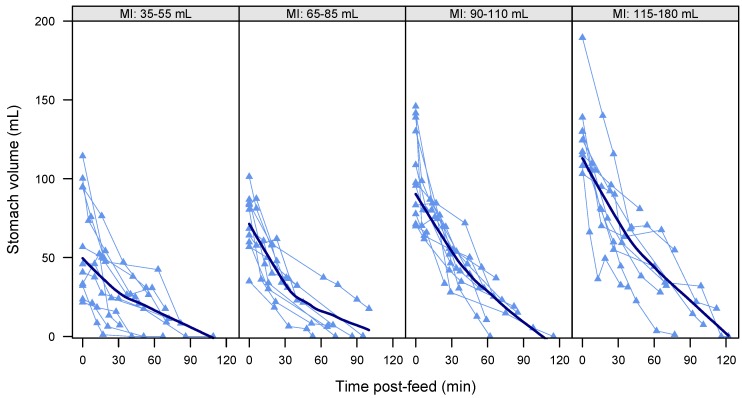
Gastric emptying of individual feeds in term breastfed infants (*n* = 41 feeds). Feeds are grouped by milk intake (MI) to illustrate the effect of the feed volumes; approximately equal numbers are included in each panel. Data points represent stomach volumes calculated from ultrasound images; connecting lines link measurements from the same feed. Bold line represents local regression smoother (LOESS, span = 0.9).

The overall decreasing curvilinear pattern of GE (linear: 0.04 [−0.17, 0.24], *p* = 0.72; square root: −10.5 [−12.7, −8.2], *p* < 0.001) is shown in Figure 2. Higher FVs were associated with faster (−0.07 [−0.10, −0.03], *p* < 0.001) GE rate (Figure 3) and higher overall post-feed stomach volumes (0.82 [0.53, 1.12], *p* < 0.001). No association was seen between feed duration and post-feed stomach volume (−0.25 [−0.68, 0.18], *p* = 0.23).

Immediate post-feed stomach volumes were not associated with R1 (*p* = 0.91).

After accounting for time post-feed, FV, and feed duration, as per the above model, larger R1 volumes (0.55 [0.24, 0.86], *p* = 0.003) and higher whey protein concentrations (4.99 [0.84, 9.13], *p* = 0.023) were associated with larger post-feed stomach volumes, while the casein:whey ratio (2.2 ± 0.88, *p* = 0.030) and lactose concentration (−0.04 ± 0.02, *p* = 0.037) modified the GE curve depending on FV. Higher casein:whey ratios at lower FVs were associated with faster GE, and at higher FVs with slower GE, while higher lactose concentrations at lower FVs were associated with slower GE, and at higher FVs with faster GE. No other associations with post-feed stomach volumes or changes to the GE curves were found (Table 3).

### 3.5. Feed Volume, Feed Duration, and Gastric Emptying during Breastfeeding

Higher FVs were associated with higher stomach volumes measured immediately post-feed (0.79 [0.51, 1.07], *p* < 0.001) and longer GE times (0.24 [0.03, 0.46], *p* = 0.033). FV was not associated with either concentrations of measured HM components or infant’s characteristics/anthropometrics/BC (Table 3).

Feed duration was not associated with FV (0.06 [−0.03, 0.15], *p* = 0.20) or R1 volume (0.01 [−0.17, 0.19], *p* = 0.91).

After accounting for R1 (1.07 [0.47, 1.7], *p* = 0.002), FV (1.00 [0.71, 1.3], *p* < 0.001) and feed duration (−0.30 [−0.96, 0.36], *p* = 0.34), immediate post-feed stomach volumes were not associated with either measured HM components (*p* > 0.068) or infant’s demographics/anthropometrics/BC (*p* > 0.46). Stomach volume reduction during breastfeeding was not associated with either measured HM components (*p* > 0.11); infant’s demographics/anthropometrics/BC (*p* > 0.48); R1, FV or feed duration (*p* > 0.34).

### 3.6. Gastric Emptying Time

The GE time was not associated with feed duration (0.35 [−0.29, 0.98], *p* = 0.28), but was negatively associated with R2 (−0.63 [−1.05, −0.21], *p* = 0.005) after accounting for FV (*p* < 0.001). Longer GE times were associated with higher adiponectin concentration (2.3 [0.9, 3.7], *p* = 0.002) and dose (0.02 [0.01, 0.03], *p* = 0.005), and lower casein:whey ratio (−65.9 [−107.1, −24.7], *p* = 0.003). No associations with infant characteristics were seen (Table 3).

### 3.7. Pre-Feed Residuals

Infants cued for F1 and F2 with different residual volumes (R1 and R2) present in their stomachs (Table 1). Larger FVs were associated with smaller R1 volumes (*p* = 0.002), with each −0.92 [−1.47, −0.37] mL of R1 volume resulting in extra mL of FV. Larger R2 volumes were associated with larger FVs (*p* = 0.006), each additional mL of FV resulting in 0.21 [0.07, 0.35] mL greater R2.

There was no association between R2 and R1 in univariate model (0.11 [−0.19, 0.42], *p* = 0.46). After accounting for FV and GE time (*p* < 0.001 for both) larger R2 volumes were associated with larger R1 volumes (0.36 [0.11, 0.60], *p* = 0.005).

After accounting for FV, R2 was not associated with any concentration of HM components (*p* ≥ 0.038 after adjusting for multiple comparisons).

### 3.8. Feeding Frequency

A longer time between the feeds was seen when infants were longer, heavier, and had higher %FM measured with BIS (Table 4) in univariate models. The associations for length and weight were not significant after accounting for the other (*p* > 0.38); the association for %FM measured with BIS was not significant after accounting for infant length (*p* = 0.095).

## 4. Discussion

Our research shows that HM components, such as adiponectin, whey protein, casein:whey ratios, lactose, total carbohydrates, and oligosaccharides are associated with gastric emptying and breastfeeding patterns of breastfed infants. GE is a mechanism involved in satiety, therefore milk components influencing GE have the potential to affect infant milk intake and therefore growth and development in early life and subsequently health later in life.

Given the assumption that HM composition potentially influences GE [14,71], in term infants we expected the appetite hormones to be associated with infant GE rate such that high concentrations and/or doses of leptin would result in slower GE [30], whereas adiponectin might induce faster GE [37] consistent with both animal and human models. However, neither the concentrations nor doses of these hormones were related to GE rate. Previously skim milk leptin was not found to be associated with either GE rate or GE time [33,34], which we have confirmed with this larger study cohort. It was speculated that whole milk leptin, which is known to be of higher concentration, might be the reason for the negative finding [32]. While our measures of whole milk leptin were typically higher, there is an opinion that values of this magnitude are unlikely to contribute considerably to infant serum levels [72] so only the local pathways would be engaged in GE regulation. As such we were unable to find a relationship between whole milk leptin and GE. This is in contrast to animal studies showing reduced GE [30] or food intake [31] after injection or oral administration of leptin, respectively. However, it is possible that the long-term energy expenditure regulatory effect of leptin [73] may mask its short-term satiety effect on GE. Alternatively, if levels of leptin are contributing significantly to serum levels, there is a possibility that the number of receptors in the stomach of the young infant is low. Further, short-term satiety signaling through hypothalamic neurons is not fully mature, both of which would allow the infant to maintain a high physiological drive to feed to ensure adequate growth [28,73]. Gender differences in infant serum leptin levels associated with adiposity [74] have also been speculated to play a role in gastric response to HM leptin, although we did not find any relationships between infant sex/adiposity and both GE rate and GE time.

In contrast to leptin, we found that increased levels and doses of adiponectin were associated with longer GE times. This finding may partially explain the growth-regulating effect of adiponectin in infants in the first six months of life [29], when high HM adiponectin concentration is associated with lower infant weight and adiposity. Further adiponectin is 20-fold higher in concentration compared to leptin and is therefore likely to have greater biological significance [75]. The lack of association between adiponectin and GE rate is in agreement with studies of rats that showed that gastric epithelium and glands are populated with adiponectin receptors, which downregulate gastric motility [36,76]. Conversely, the findings are in contrast to studies of type 2 diabetic adults, in which elevated levels of adiponectin were associated with faster GE [37]. Further, other HM hormones such as ghrelin, cholecystokinin, and insulin may counteract or interact synergistically with leptin [77,78] and/or adiponectin.

Our study examined an extensive array of macronutrients beyond fat, total protein and lactose. Consistent with the findings of Cannon et al. [34] there were no associations with fat and total protein and either GE rate or GE time. Studies in dogs indicate that all three major macronutrients activate the ileal brake, resulting in reduction of GE; limited human studies support the findings for fat and carbohydrates while associations with protein are not so straightforward [79,80]. We were unable to find associations between the HM fat content and GE consistent with findings of Khan et al. [39] and Kent et al. [38] regarding the feeding frequency. This may be because lipids initiate the ileal brake when they reach the ileum via hydrolysis of triacylglycerol into fatty acids, thereby producing a delay in GE in humans [79]. Further analysis of HM fatty acids may shed more light on GE in breastfed infants.

However, we have found that higher whey protein concentrations are associated with larger post-feed stomach volumes, although we did not see any interaction with time, so no effect on GE rate was detected. This contradicts the results of studies of GE conducted on breastfed and formula-fed infants or studies of formula with different casein:whey ratio [15,81] in which a fast or slow GE rate was explained by concentrations of whey protein or casein, respectively. Previous studies, however, could not adequately analyze the effect of the whey protein concentration in conjunction with volume, as they only reported gastric half-emptying time, restricted monitoring time, and/or controlled infants’ volume intakes. The whey fraction of HM is highly soluble in the gastric juices and rapidly empties from the stomach compared to other proteins such as casein. Whey isolate, however, was associated with a lower gastric inhibitory polypeptide (GIP) response in adults, consistent with decreased rate of GE [82]. It may very well be that whey protein speeds up the initial stage of GE (probably during the breastfeeding time), but once it activates jejunal or ileal brakes the overall GE is reduced.

While lactose is related to GE rate, it is affected by FV; at the middle range FVs (71–108 mL) lactose has no relationship with GE, whereas at lower FVs higher lactose concentrations are associated with slower GE, and at higher FVs with faster GE. These results are consistent with Khan et al. [39], who reported an association of higher lactose concentration with increased feeding frequency. These findings could be an important addition to the evaluation of the digestive and metabolic effect of lower breastfeeding frequency and larger FVs, common in Western countries, contrary to the lactation practices in traditional societies [83].

In terms of casein:whey ratios the effect is opposite to that of lactose where at lower FVs higher casein:whey ratios are associated with faster GE, and at higher FVs with slower GE, which may explain the contradictory findings for casein associations with GE rate in previous studies [14,23]. Cows’ milk casein was found to activate the ileal brake in adults, resulting in reduced food intake, although its effect on GE was not significant [84]. The finding of smaller volumes resulting in more rapid GE rate might be explained by the time casein spends in the acidic environment of the stomach. While soluble whey proteins rapidly enter the small intestine mostly intact, casein transit is delayed due to the curd formation. When it exits the stomach it is mainly in the form of degraded peptides [85]. If the FVs are small some casein may exit intact, thereby speeding up GE, while if the FVs are large, casein curdles and degrades to the opioid peptides that slow down GE [86]. However, this mechanism does not explain why higher casein:whey ratios of HM were associated with shorter GE time, which could be due to the smaller amounts of whey protein reaching the small intestine and having less effect on jejunal or ileal brakes [80]. Our finding that higher whey protein concentrations are associated with larger post-feed stomach volumes further supports this possible explanation.

Further, *k*-casein has been shown to inhibit the binding of *Helicobacter pylori* to human mucosa in vitro [87]. *Helicobacter pylori* are Gram-negative bacteria present in the stomach, and are known to downregulate levels of ghrelin and leptin in the stomach [88], which may significantly affect GE. The protective action of HM *k*-casein is reinforced by lysozyme, one of the major whey proteins. While we have not seen any significant associations between lysozyme and GE, lysozyme contributes to the control of the GI bacterial population [89], and could be upregulated to control the bacterial population in the GI tract [90] and increase digestion of microbial protein [24], all of which could potentially influence GE. In a clinical study of preterm infants, lysozyme added to donor HM or formula was associated with increased body weight, normalization of the stool, and improved feed tolerance [91]. While all of this suggests that lysozyme could potentially have an effect on GE in certain circumstances, given that we have studied a healthy population the magnitude of the effect could be insignificant.

GE during feed administration has been previously documented in preterm infants. In this study, an average of 20% of feed volume is emptied from the stomach during breastfeeding compared with 10% in preterm [92]. This is probably due to a more mature GI tract in term infants and the effect of both larger FVs and present pre-feed residuals, which were associated with faster GE rate, but not to the longer feed duration time in term infants or milk composition as no associations were found.

While we speculated that milk composition might regulate the milk intake of the infant and/or the residual volume in the stomach prior to cueing for the next feed, we were unable to show this. Rather, FV is more strongly associated with GE rate than variations in milk composition. Gastric mechanosensation is an important factor in the regulation of satiation during food intake. Indeed, gastric distention is an important determinant of GE [93], and volume-related suppression of GE rate has been reported in animal models [94]. The observed volume-related acceleration of GE with larger FVs emptying more quickly in term breastfed infants is consistent with our previous findings [34]. The biggest effect of volume was seen after the feed and as the post-prandial period progressed the magnitude of this effect decreased (Figure 2). This may also explain the variability in the time between each feed for an infant over a 24-h period [33,38]. Feeding frequency decreases between one and three months of lactation, while milk intake during each breastfeeding session increases, with both parameters remaining constant up to six months [95]. This is attributed to the fact that as infants mature they become able to consume larger FVs [38], resulting in a longer time between feeds. Also, larger FVs are generally consumed at night or in the early morning when the frequency of feeding declines [33,38]. This decline in feeding frequency also coincides with higher nocturnal concentrations of leptin and fat, and lower concentrations of lactose in HM [33], although relationships between both feeding frequency and FV and these components’ concentrations are yet to be evaluated.

The recommendations for breastfeeding are to feed on demand. Interestingly, we found that the majority of infants cued for a feed when milk was still present in the stomach, albeit in variable volumes (Table 1). This suggests that the reduction of gastric distension, which regulates hunger sensations, plays a greater role in signaling time to feed [96]. Further, it may be beneficial to the developing infant to have the gastric mucosa exposed to HM anti-inflammatory components such as lysozyme or immunomodulatory agents and growth factors, all of which contribute to the maturation of the GI tract [8]. Thus it may be detrimental to prescribe decreasing the frequency of feeding in breastfed infants or expect the infant stomach to be empty in order to feed again [97].

Furthermore, interesting associations were observed between infant milk intake and volumes remaining in the stomach prior to the first and second feed. Smaller residual volumes prior to the first feed were associated with greater milk intakes, and greater milk intakes were associated with larger volumes in the stomach prior to feeding again. This suggests that breastfed infants may appear to be consuming HM volumes in a variable pattern, but due to varying residuals may actually be feeding to a predetermined stomach volume, which is also supported by the positive relationship between both pre-feed residuals (R1, R2). In fasting adults ghrelin was found to increase and spontaneously decrease at the time points of the customary meals [98], supporting the involvement of the brain in GI tract regulation. Further studies monitoring two or more consecutive feeds or even 24-h GE measurements and analyses of ghrelin in HM would clarify this finding.

In healthy adults post-lag GE and colonic transfer is reported to be faster in men than in women [99]. In this study infant sex, age, anthropometrics, and BC were not associated with GE and breastfeeding parameters, with the exception of feeding frequency. Feeding frequency decreases in the first three months of lactation and then remains stable until six months [95,100]. The absence of a significant association between feeding frequency and age, together with associations with anthropometric and body composition parameters, illustrates that feeding frequency is dictated by the growth and development of an infant rather than the infant age. These findings further underline the need for breastfeeding on demand, with the frequency linked to individual infant growth rates rather than scheduled feeding, which could exert a detrimental effect on infant growth.

While the monitoring of a single feed limits the analysis possibilities, examination of multiple feeds requires the study to be carried out in the mother’s home for long periods of time. The sample size is not a limitation of the study, as although no associations between milk composition and GE rate were detected, we were able to clearly show a relationship between FV and GE rate as well as associations between milk composition and other GE parameters.

## 5. Conclusions

Human milk appetite hormones and macronutrients and feed volume affect gastric emptying and feeding patterns in term breastfed infants. Adiponectin, whey protein, and casein:whey ratio are associated with GE, while the effects of casein:whey ratios and lactose concentrations on GE vary with feed volume. Larger feed volumes result in a faster GE rate. Thus, milk composition and feed volume play an important role in appetite regulation via gastric function.

## Figures and Tables

**Table 1:** Participant characteristics expressed as mean ± SD and range.

Characteristics	2 mo ^a^		5 mo ^b^		Total ^c^	
	Mean ± SD	Range	Mean ± SD	Range	Mean ± SD	Range
*Infant characteristics*						
Infant age (weeks)	9 ± 1	6–10	22 ± 1	18–23	16 ± 7	6–23
Infant length (cm)	57 ± 2	53–61	65 ± 2 ***	62–69	61 ± 4	53–69
Infant weight (kg)	5.3 ± 0.8	4.2–6.3	7.2 ± 1.0 ***	5.8–9.5	6.3 ± 1.3	4.2–9.5
Infant BMI	15.9 ± 1.3	13.9–18.1	17.6 ± 1.7 ***	14.9–20.4	16.7 ± 1.7	13.9–20.4
HC (cm)	39 ± 1	37–42	43 ± 2 ***	40–46	41 ± 2	37–46
Fat Mass with BIS (%)	21.4 ± 3.6	11.1–27.1	28.9 ± 3.2 ***	21.7–35.8	25.3 ± 5.0	11.1–35.8
Fat Mass with US (%)	24.2 ± 3.6	17.5–30.5	26.6 ± 3.6	20.8–35.9	25.5 ± 3.8	17.5–35.9
*BF/GE characteristics*						
Feed volume (mL)	86 ± 34	35–140	85 ± 33	36–180	86 ± 33	35–180
SV after feed 1 (mL)	87 ± 36	32–141	93 ± 41	22–189	90 ± 38	22–189
Feed duration (min)	28 ± 14	11–72	20 ± 8	6–37	24 ± 12	6–72
SV reduction (mL) ^d^	5 ± 21	(−42)–33	4 ± 26	(−57)–56	4 ± 24	(−57)–56
GE time (min) ^e^	94 ± 29	44–153	88 ± 18	50–140	91 ± 24	44–140
Residual 1 (mL)	6 ± 12	0–50	11 ± 19	0–62	9 ± 16	0–62
Residual 2 (mL)	20 ± 20	0–81	15 ± 15	0–55	18 ± 18	0–81
Feeding frequency (h) ^f^	2.3 ± 0.7	1.0–4.0	2.7 ± 0.8	1.5–4.0	2.5 ± 0.7	1.0–4.0

Data are mean ± SD and ranges. ^a^ *n* = 20; ^b^ *n* = 21. ^c^ *n* = 41 feeds. ^d^ Stomach volume reduction during feed time is calculated as the difference between the sum of residual 1 and feed volume and the immediate stomach volume after Feed 1 . ^e^ GE time is the time from the start of Feed 1 to the start of Feed 2 (time between feeds plus feed duration). ^f^ Feeding frequency self-reported by mothers as to how often infant feeds (e.g., every three hours). *** Indicates significant differences (*p* < 0.001) between two- and five-month-old infants. Abbreviations: BF—breastfeeding; BIS—bioimpedance spectroscopy; GE—gastric emptying; HC—head circumference; SV—stomach volume; US—ultrasound.

**Table 2:** Concentrations and doses of measured HM hormones and macronutrients.

Components	Concentration		Dose Per Feed	
	Mean ± SD	Range	Mean ± SD	Range
Adiponectin (ng/mL, ng)	10.02 ± 4.08	6.18–22.58	868.62 ± 491.32	238.60–2536.91
WM leptin (ng/mL, ng)	0.51 ± 0.18	0.23–1.10	44.80 ± 24.30	10.15–115.03
SM leptin (ng/mL, ng)	0.28 ± 0.12	0.20–0.84	24.8 ± 15.0	6.91–73.00
Total protein (g/L, g)	11.29 ± 2.56	7.60–24.16	0.99 ± 0.39	0.35–2.29
Casein (g/L, g)	1.54 ± 0.53	0.69–3.45	0.14 ± 0.07	0.04–0.29
Whey protein (g/L, g)	5.97 ± 1.26	3.82–9.08	0.52 ± 0.19	0.17–0.95
Casein:whey ratio	0.27 ± 0.11	0.10–0.73	n/a ^a^	n/a ^a^
Lysozyme (g/L, g)	0.14 ± 0.12	0.05–0.48	0.01 ± 0.01	0.003–0.030
TCH (g/L, g)	82.72 ± 7.89	67.08–97.49	7.28 ± 2.62	3.28–15.18
Lactose (g/L, g)	65.84 ± 5.14	53.49–77.94	5.86 ± 2.22	2.19–12.06
HMO (g/L, g)	16.88 ± 9.89	(−10.86) ^b^–35.77	1.42 ± 0.94	(−1.09) ^b^–3.78
Fat (g/L, g)	42.74 ± 12.10	17.42–66.79	3.57 ± 1.45	0.64–6.40

Data are mean ± SD and ranges, *n* = 41 feeds. ^a^ Casein:whey ratios for doses are the same as for concentrations. ^b^ Negative values are seen for human milk oligosaccharides (HMO) when lactose measurements are higher than total carbohydrates. Abbreviations: SM—skim milk; TCH—total carbohydrates; WM—whole milk.

**Table 3:** HM components and infant characteristics and their associations with feed variables and gastric emptying.

Predictors	Feed Volume ^a^		Gastric Emptying Time ^a^		Post-Feed Stomach Volumes ^b^	
	Estimate ± SE (95% CI)	*p*-Value	Estimate ± SE (95% CI)	*p*-Value	Estimate ± SE (95% CI)	*p*-Value
***Concentrations***						
Adiponectin (ng/mL)	1 ± 1.3 (−36.6, 134.5)	0.44	2.3 ± 0.7 (0.9, 3.7)	**0.002** ^c^	1.3 ± 0.7 (−0.2, 2.7)	0.081
Whole milk leptin (ng/mL)	9.9 ± 29.9 (−36.6, 134.5)	0.74	6.8 ± 15.8 (−24.2, 37.8)	0.67	−9.5 ± 13.3 (−35.8, 16.9)	0.48
Skim milk leptin (ng/mL)	49 ± 43.6 (−36.6, 134.5)	0.26	6.7 ± 24.3 (−41, 54.4)	0.78	39.8 ± 18.4 (−0.8, 80.3)	0.054
Total protein (g/L)	−2.1 ± 2.1 (−6.2, 1.9)	0.30	−0.9 ± 1.1 (−3.1, 1.3)	0.41	1.1 ± 1 (−1.2, 3.4)	0.30
Whey protein (g/L)	−5.5 ± 4.2 (−13.8, 2.7)	0.19	5.8 ± 2.2 (1.6, 10.1)	0.011	5 ± 1.9 (0.8, 9.1)	**0.023**
Casein (g/L)	2.6 ± 10.2 (−17.4, 22.5)	0.80	−12.4 ± 4.7 (−21.5, −3.2)	0.013	−2 ± 4.4 (−11.6, 7.6)	0.66
Casein:whey ratio	24.5 ± 46.1 (−65.9, 114.9)	0.59	−65.9 ± 21 (−107.1, −24.7)	**0.003**	−17.3 ± 20.1 (−61.4, 26.9)	0.41
Lysozyme (g/L)	−81.4 ± 46.2 (−172, 9.1)	0.079	−19.5 ± 28.3 (−75, 36)	0.49	23.3 ± 15.4 (−7.2, 53.8)	0.13
Total carbohydrates (g/L)	−1.1 ± 0.7 (−2.3, 0.2)	0.12	−0.6 ± 0.4 (−1.3, 0.1)	0.10	−0.5 ± 0.3 (−1.2, 0.1)	0.089
Lactose (g/L)	0.7 ± 1.1 (−1.4, 2.7)	0.51	0.2 ± 0.6 (−0.9, 1.3)	0.76	0.03 ± 0.49 (−1, 1.1)	0.96
HMO (g/L)	−0.8 ± 0.5 (−1.9, 0.2)	0.13	−0.4 ± 0.3 (−1, 0.2)	0.16	−0.4 ± 0.2 (−0.9, 0.1)	0.13
Fat (g/L)	−0.69 ± 0.6 (−1.8, 0.5)	0.26	−0.1 ± 0.3 (−0.6, 0.5)	0.79	−0.1 ± 0.3 (−0.9, 0.6)	0.71
***Doses***						
Adiponectin (ng)	n/a ^d^	n/a ^d^	0.02 ± 0.01 (0.01, 0.03)	**0.005**	0.01 ± 0.01 (−0.003, 0.03)	0.094
Whole milk leptin (ng)	n/a	n/a	−0.1 ± 0.2 (−0.4, 0.2)	0.44	−0.2 ± 0.2 (−0.5, 0.2)	0.28
Skim milk leptin (ng)	n/a	n/a	−0.2 ± 0.2 (−0.7, 0.3)	0.38	0.4 ± 0.2 (−0.1, 0.8)	0.086
Total protein (g)	n/a	n/a	−25.9 ± 12.4 (−50.2, −1.7)	0.040	15 ± 13.1 (−13.7, 43.7)	0.27
Whey protein (g)	n/a	n/a	47.6 ± 18.7 (10.8, 84.3)	0.015	50.6 ± 24.3 (−2.8, 104)	0.061
Casein (g)	n/a	n/a	−119 ± 53.3 (−223.4, −14.6)	0.030	0.4 ± 47.6 (−104.2, 105.1)	0.99
Lysozyme (g)	n/a	n/a	−276.2 ± 370.7 (−1002.9, 450.4)	0.46	395.5 ± 258.3 (−114.7, 905.6)	0.13
Total carbohydrates (g)	n/a	n/a	−4.1 ± 1.8 (−7.6, −0.5)	0.030	−4.6 ± 3 (−11.2, 2.1)	0.16
Lactose (g)	n/a	n/a	−5.8 ± 2.7 (−11.1, −0.6)	0.037	−3.1 ± 5.4 (−15.1, 8.9)	0.58
HMO (g)	n/a	n/a	−3.1 ± 3.1 (−9.2, 3)	0.32	−3.2 ± 2.7 (−9.1, 2.8)	0.27
Fat (g)	n/a	n/a	−2.8 ± 2.7 (−8.1, 2.4)	0.30	−4.9 ± 2.8 (−12.7, 2.8)	0.15
***Demographics***						
Infant sex (Male)	−2.2 ± 10.7 (−23.1, 18.8)	0.84	−1.5 ± 7.5 (−16.3, 13.2)	0.84	−8.4 ± 4.6 (−17.8, 1.1)	0.081
Infant age (months)	−0.9 ± 3.6 (−7.9, 6)	0.80	−1.8 ± 2.5 (−6.6, 3)	0.47	−1.5 ± 1.4 (−4.5, 1.6)	0.32
***Anthropometrics***						
Infant length (cm)	−0.03 ± 1.3 (−2.6, 2.6)	0.98	−1.3 ± 0.9 (−3, 0.4)	0.15	−0.5 ± 0.6 (−1.8, 0.8)	0.44
Infant weight (kg)	0.7 ± 4.1 (−7.4, 8.8)	0.87	−2.3 ± 2.9 (−7.9, 3.4)	0.43	−2.3 ± 1.8 (−6.3, 1.7)	0.23
Head circumference (cm)	−2.5 ± 2.6 (−7.5, 2.5)	0.34	−1.4 ± 1.8 (−4.9, 2.1)	0.42	−1.8 ± 1.2 (−4.5, 0.8)	0.15
Infant BMI	−0.2 ± 3.2 (−6.5, 6)	0.94	−1.5 ± 2.2 (−5.8, 2.8)	0.48	−3.2 ± 1.5 (−6.6, 0.2)	0.062
***Body composition***						
Fat mass with US (%)	0.6 ± 1.4 (−2.2, 3.4)	0.67	−0.3 ± 0.9 (−2.2, 1.5)	0.71	−0.6 ± 0.7 (−2.1, 1.0)	0.42
Fat mass with BIS (%)	0.4 ± 1.1 (−1.8, 2.5)	0.74	−0.4 ± 0.7 (−1.9, 1)	0.56	−0.5 ± 0.5 (−1.5, 0.5)	0.35

Data are parameter estimate ± SE and 95% CI, *n* = 41 feeds. ^a^ Effects of predictors taken from univariate regression models; ^b^ Effects of predictors taken from linear mixed effects models that accounted for postprandial time, feed volume and feed duration. ^c^ After the false discovery rate adjustment the *p*-values were considered to be significant at <0.011 for GE time (bold font); ^d^ n/a—dosage is dependent on feed volume. Abbreviations: BIS—bioimpedance spectroscopy; HMO—human milk oligosaccharides; US—ultrasound skinfolds.

**Table 4:** Associations between infant feeding frequency and HM components and infant characteristics.

Predictors	Feeding Frequency (h) ^a^	
	Estimate ± SE (95% CI) ^b^	*p*-Value
*Concentrations*		
Adiponectin (ng/mL)	−0.001 ± 0.03 (−0.06, 0.06)	0.96
Whole milk leptin (ng/mL)	−1.1 ± 0.7 (−2.5, 0.3)	0.13
Skim milk leptin (ng/mL)	0.8 ± 1.6 (−2.3, 4)	0.60
Total protein (g/L)	−0.05 ± 0.05 (−0.15, 0.04)	0.28
Whey protein (g/L)	−0.1 ± 0.1 (−0.3, 0.1)	0.42
Casein (g/L)	0.04 ± 0.2 (−0.4, 0.5)	0.86
Casein:whey protein ratio	0.4 ± 1.1 (−1.7, 2.5)	0.68
Lysozyme (g/L)	−0.4 ± 1.1 (−2.5, 1.7)	0.71
Total carbohydrates (g/L)	0.01 ± 0.02 (−0.03, 0.04)	0.73
Lactose (g/L)	−0.05 ± 0.02 (−0.1, −0.01)	0.031
HMO (g/L)	0.01 ± 0.02 (−0.03, 0.04)	0.73
Fat (g/L)	−0.02 ± 0.01 (−0.04, 0.01)	0.19
*Doses*		
Adiponectin (ng/mL)	0.0002 ± 0.0003 (−0.0004, 0.0008)	0.50
Whole milk leptin (ng/mL)	−0.002 ± 0.01 (−0.01, 0.01)	0.80
Skim milk leptin (ng/mL)	0.01 ± 0.01 (−0.01, 0.03)	0.59
Total protein (g/L)	0.1 ± 0.3 (−0.5, 0.8)	0.67
Whey protein (g/L)	0.1 ± 0.7 (−1.3, 1.5)	0.89
Casein (g/L)	2.1 ± 2 (−1.7, 5.9)	0.27
Lysozyme (g/L)	−5.7 ± 17.2 (−39.4, 27.9)	0.73
Total carbohydrates (g/L)	0.1 ± 0.1 (0, 0.2)	0.22
Lactose (g/L)	0.04 ± 0.06 (−0.08, 0.17)	0.49
HMO (g/L)	0.3 ± 0.1 (0, 0.5)	0.051
Fat (g/L)	−0.2 ± 0.1 (−0.5, 0)	0.085
*Demographics*		
Infant sex (Male)	−0.2 ± 0.3 (−0.7, 0.4)	0.53
Infant age (months)	0.2 ± 0.1 (0, 0.3)	0.078
*Anthropometrics*		
Infant length (cm)	0.1 ± 0.03 (0.04, 0.15)	**0.004 ^c^**
Infant weight (kg)	0.2 ± 0.1 (0.1, 0.4)	**0.010**
Head circumference (cm)	0.1 ± 0.1 (0, 0.2)	0.23
Infant BMI	0.13 ± 0.1 (0, 0.3)	0.10
*Body composition*		
% fat mass with US	0.07 ± 0.03 (0, 0.13)	0.040
% fat mass with BIS	0.08 ± 0.02 (0.03, 0.12)	**0.002**

Data are parameter estimate ± SE and 95% CI, *n* = 41 feeds. ^a^ Feeding frequency self-reported by mothers as to how often infant feeds (e.g., every three hours). ^b^ Effects of predictors are results of univariate regression model. ^c^ After the false discovery rate adjustment the *p*-values were considered to be significant at <0.031 (highlighted). Abbreviations: BIS—bioimpedance spectroscopy; HMO—human milk oligosaccharides; US—ultrasound skinfolds.

## References

[1] Geddes D., Prescott S. (2013). Developmental origins of health and disease: The role of human milk in preventing disease in the 21(st) century. J. Hum. Lact..

[2] Marseglia L., Manti S., D’Angelo G., Cuppari C., Salpietro V., Filippelli M., Trovato A., Gitto E., Salpietro C., Arrigo T. (2015). Obesity and breastfeeding: The strength of association. Women Birth.

[3] Savino F., Liguori S., Fissore M., Oggero R. (2009). Breast milk hormones and their protective effect on obesity. Int. J. Pediatr. Endocrinol..

[4] Bartok C. (2011). Babies fed breastmilk by breast versus by bottle: A pilot study evaluating early growth patterns. Breastfeed. Med..

[5] Sievers E., Oldigs H.D., Santer R., Schaub J. (2001). Feeding patterns in breast-fed and formula-fed infants. Ann. Nutr. Metab..

[6] Le Huërou-Luron I., Bouzerzour K., Ferret-Bernard S., Ménard O., Le Normand L., Perrier C., Le Bourgot C., Jardin J., Bourlieu C., Carton T. (2016). A mixture of milk and vegetable lipids in infant formula changes gut digestion, mucosal immunity and microbiota composition in neonatal piglets. Eur. J. Nutr..

[7] Manti S., Lougaris V., Cuppari C., Tardino L., Dipasquale V., Arrigo T., Salpietro C., Leonardi S. (2016). Breastfeeding and il-10 levels in children affected by cow’s milk protein allergy: A restrospective study. Immunobiology.

[8] Goldman A.S. (2000). Modulation of the gastrointestinal tract of infants by human milk. Interfaces and interactions. An evolutionary perspective. J. Nutr..

[9] Hanson C., Lyden E., Furtado J., Van Ormer M., Anderson-Berry A. (2016). A comparison of nutritional antioxidant content in breast milk, donor milk, and infant formulas. Nutrients.

[10] Hunt J. (1980). A possible relation between the regulation of gastric emptying and food intake. Am. J. Physiol..

[11] Hellstrom P., Gryback P., Jacobsson H. (2006). The physiology of gastric emptying. Best Pract. Res. Clin. Anaesthesiol..

[12] Gomez H., Hornoy P., Liehn J. (2003). Ultrasonography and gastric emptying in children: Validation of a sonographic method and determination of physiological and pathological patterns. Pediatr. Radiol..

[13] Carlos M., Babyn P., Marcon M., Moore A. (1997). Changes in gastric emptying in early postnatal life. J. Pediatr..

[14] Perrella S., Hepworth A., Simmer K., Geddes D. (2015). Influences of breast milk composition on gastric emptying in preterm infants. J. Paediatr. Gastroenterol. Nutr..

[15] Cavell B. (1979). Gastric emtying in preterm infants. Acta Paediatr. Scand..

[16] Armand M., Hamosh M., Mehta N., Angelus P., Rhilpott J., Henderson T., Dwyer N., Lairon D., Hamosh P. (1996). Effect of human milk or formula on gastric function and fat digestion in the premature infant. Pediatr. Res..

[17] Cavell B. (1981). Gastric emptying in infants fed human milk or infant formula. Acta Paediatr. Scand..

[18] Vist G., Maughan R. (1995). The effect of osmolality and carbohydrate content on the rate of gastric emptying of liquids in man. J. Physiol..

[19] Hunt L., Antonson D., Paxson C.J., Vanderhoff J. (1982). Osmolality of carbohydrate solutions and gastric emptying in the newborn. Am. J. Dis. Child..

[20] Husband J., Husband P., Mallinson C. (1970). Gastric emptying of starch meals in the newborn. Lancet.

[21] Lonnerdal B. (2010). Bioactive proteins in human milk: Mechanisms of action. J. Pediatr..

[22] Michaelsen K., Larnkjaer A., Molgaard C. (2012). Amount and quality of dietary proteins during the first two years of life in relation to NCD risk in adulthood. Nutr. Metab. Cardiovasc. Dis..

[23] Billeaud C., Guillet J., Sandler B. (1990). Gastric emptying in infants with or without gastro-oesophageal reflux according to type of milk. Eur. J. Clin. Nutr..

[24] Wang G., Lo L., Forsberg L., Maier R. (2012). Helicobacter pylori peptidoglycan modifications confer lysozyme resistance and contribute to survival in the host. mBio.

[25] Artym J., Zimecki M. (2013). Milk-derived proteins and peptides in clinical trials. Postepy Hig. Med. Dosw..

[26] Savino F., Sardo A., Rossi L., Benetti S., Savino A., Silvestro L. (2016). Mother and infant body mass index, breast milk leptin and their serum leptin values. Nutrients.

[27] Wang Y.Y., Zhang Z.J., Yao W., Morrow A., Peng Y.M. (2011). Variation of maternal milk adiponectin and its correlation with infant growth. Zhonghua Er Ke Za Zhi.

[28] Miralles O., Sanchez J., Palou A., Pico C. (2006). A physiological role of breast milk leptin in body weight control in developing infants. Obesity.

[29] Woo J., Guerrero M., Altaye M., Ruiz-Palacios G., Martin L., Dubert-Ferrandon A., Newburg D., Morrow A. (2009). Human milk adiponectin is associated with growth in two independent cohorts. Breastfeed. Med..

[30] Smedh U., Hakansson M.L., Meister B., Uvnas-Moberg K. (1998). Leptin injected into the fourth ventricle inhibits gastric emptying. Neuroreport.

[31] Sanchez J., Oliver P., Miralles O., Ceresi E., Pico C., Palou A. (2005). Leptin orally supplied to neonate rats is directly uptaken by the immature stomach and may regulate short-term feeding. Endocrinology.

[32] Kugananthan S., Lai C.T., Gridneva Z., Mark P.J., Geddes D.T., Kakulas F. (2016). Leptin levels are higher in whole compared to skim human milk, supporting a cellular contribution. Nutrients.

[33] Cannon A., Kakulas F., Hepworth A., Lai C., Hartmann P., Geddes D. (2015). The effects of leptin on breastfeeding behaviour. Int. J. Environ. Res. Public Helath.

[34] Cannon A.M., Gridneva Z., Hepworth A., Lai C.T., Tie W.J., Khan S., Hartmann P.E., Geddes D.T. (2016). The relationship of human milk leptin and macronutrients with gastric emptying in term breastfed infants. Pediatr. Res..

[35] Newburg D., Woo J., Morrow A. (2010). Characteristics and potential functions of human milk adiponectin. J. Pediatr..

[36] Kentish S.J., Ratcliff K., Li H., Wittert G.A., Page A.J. (2015). High fat diet induced changes in gastric vagal afferent response to adiponectin. Physiol. Behav..

[37] Iwase M., Iino K., Oku M., Nohara S., Asano T., Doi Y., Iida M. (2009). Serum high-molecular weight adiponectin is related to early postprandial glycemic increases and gastric emptying in patients with type 2 diabetes mellitus. Diabetes Metab. Res. Rev..

[38] Kent J.C., Mitoulas L.R., Cregan M.D., Ramsay D.T., Doherty D.A., Hartmann P.E. (2006). Volume and frequency of breastfeedings and fat content of breast milk throughout the day. Pediatrics.

[39] Khan S., Hepworth A.R., Prime D.K., Lai C.T., Trengove N.J., Hartmann P.E. (2013). Variation in fat, lactose, and protein composition in breast milk over 24 h: Associations with infant feeding patterns. J. Hum. Lact..

[40] Gridneva Z., Hepworth A., Ward L., Lai C.T., Hartmann P., Geddes D.T. (2016). Bioimpedance spectroscopy in the infant: Effect of milk intake and extracellular fluid reservoirs on resistance measurements in term breastfed infants. Eur. J. Clin. Nutr..

[41] Gridneva Z., Hepworth A.R., Ward L.C., Lai C.T., Hartmann P.E., Geddes D.T. (2016). Determinants of body composition in breastfed infants using bioimpedance spectroscopy and ultrasound skinfolds—Methods comparison. Pediatr. Res..

[42] Arthur P., Hartmann P., Smith M. (1987). Measurement of the milk intake of breast-fed infants. J. Paediatr. Gastroenterol. Nutr..

[43] Neville M.C., Keller R., Seacat J., Lutes V., Neifert M., Casey C., Allen J., Archer P. (1988). Studies in human lactation: Milk volumes in lactating women during the onset of lactation and full lactation. Am. J. Clin. Nutr..

[44] Perrella S., Hepworth A., Simmer K., Geddes D. (2013). Validation of ultrasound methods to monitor gastric volume changes in preterm infants. J. Paediatr. Gastroenterol. Nutr..

[45] Fleet I., Linzell J. (1964). A rapid method of estimating fat in very small quantities of milk. J. Physiol..

[46] Meier P., Engstrom J., Zuleger J., Motykowski J., Vasan U., Meier W., Hartmann P.E., Williams T.M. (2006). Accuracy of a user-friendly centrifuge for measuring creamatocrits on mothers’ milk in the clinical setting. Breastfeed. Med..

[47] Mitoulas L.R. (2000). Short- and Long-Term Variation in the Production, Content and Composition of Human Milk Fat. Ph.D. Thesis.

[48] Keller R., Neville M. (1986). Determination of total protein in human milk: Comparison of methods. Clin. Chem..

[49] Kunz C., Lonnerdal B. (1989). Human milk proteins: Separation of whey proteins and their analysis by polyacrylamide gel electrophoresis, fast protein liquid chromatography (FPLC) gel filtration, and anion-exchange chromatography. Am. J. Clin. Nutr..

[50] Khan S., Casadio Y., Lai C., Prime D., Hepworth A., Trengove N., Hartmann P. (2012). Investigation of short-term variations in casein and whey proteins in breast milk of term mothers. Hepatol. Nutr..

[51] Mitoulas L.R., Kent J.C., Cox D.B., Owens R.A., Sherriff J.L., Hartmann P.E. (2002). Variation in fat, lactose and protein in human milk over 24 h and throughout the first year of lactation. Br. J. Nutr..

[52] Selsted M., Martinez R. (1980). A simple and ultrasensitive enzymatic assay for the quantitative determination of lysozyme in the picogram range. Anal. Biochem..

[53] Euber J., Brunner J. (1979). Determination of lactose in milk products by high-performance liquid chromatography. J. Dairy Sci..

[54] Albalasmeh A., Berhe A., Ghezzehei T. (2013). A new method for rapid determination of carbohydrate and total carbon concentrations using UV spectrophotometry. Carbohydr. Polym..

[55] Kuhn N., Lowenstein J. (1967). Lactogenesis in the rat. Changes in metabolic parameters at parturition. Biochem. J..

[56] Newburg D., Neubauer S., Jensen R. (1995). Carbohydrates in milks: Analysis, quantities, and significance. Handbook of Milk Composition.

[57] Lingwood B., Van Leeuwen A., Carberry A., Fitzgerald E., Callaway L., Colditz P., Ward L. (2012). Prediction of fat-free mass and percentage of body fat in neonates using biolelectrical impedance analysis and anthropometric measures: Validation against pea pod. Br. J. Nutr..

[58] Brook C. (1971). Determination of body composition of children from skinfold measurements. Arch. Dis. Child..

[59] Lohman T., Boileau R.A. (1984). Body composition in children and youth. Advances in Pediatric Sport Sciences.

[60] R Core Team (2009). R: A Language and Environment for Statistical Computing.

[61] Pinheiro J., Bates D., DebRoy S., Sarkar D., Team T.R.C. (2016). nlme: Linear and Nonlinear Mixed Effects Models. http://CRAN.R-project.org/package=nlme.

[62] Sarkar D. (2008). Lattice: Multivariate Data Visualization with R.

[63] Sarkar D., Andrews F. (2016). LatticeExtra: Extra Graphical Utilities Based on Lattice.

[64] Fox J., Weisberg S. (2011). An R Companion to Applied Regression.

[65] Venables W.N., Ripley B.D. (2002). Modern Applied Statistics with S.

[66] Maechler M. (2016). Sfsmisc: Utilities from Seminar Fuer Statistik Eth Zurich. http://cran.r-project.org/package=sfsmisc.

[67] Hothorn T., Bretz F., Westfall P. (2008). Simultaneous inference in general parametric models. Biom. J..

[68] Cannon A.M., Sakalidis V.S., Lai C.T., Perrella S.L., Geddes D.T. (2016). Vacuum characteristics of the sucking cycle and relationships with milk removal from the breast in term infants. Early Hum. Dev..

[69] Royston P., Altman D.G. (1994). Regression using fractional polynomials of continuous covariates: Parsimonious parametric modelling. J. R. Stat. Soc. Ser. C Appl. Stat..

[70] Curran-Everett D. (2000). Multiple comparisons: Philosophies and illustrations. Am. J. Physiol. Regul. Integr. Comp. Physiol..

[71] Meyer R., Foong R., Thapar N., Kritas S., Shah N. (2015). Systematic review of the impact of feed protein type and degree of hydrolysis on gastric emptying in children. BMC Gastroenterol..

[72] Lonnerdal B., Havel P.J. (2000). Serum leptin concentrations in infants: Effects of diet, sex, and adiposity. Am. J. Clin. Nutr..

[73] Bouret S.G., Draper S.J., Simerly R.B. (2004). Trophic action of leptin on hypothalamic neurons that regulate feeding. Science.

[74] Petridou E., Mantzoros C.S., Belechri M., Skalkidou A., Dessypris N., Papathoma E., Salvanos H., Lee J.H., Kedikoglou S., Chrousos G. (2005). Neonatal leptin levels are strongly associated with female gender, birth length, IGF-I levels and formula feeding. Clin. Endocrinol..

[75] Savino F., Benetti S., Liguori S., Sorrenti M., Cordero Di Montezemolo L. (2013). Advances of human milk hormones and protection against obesity. Cell. Mol. Biol..

[76] Gonzalez C.R., Caminos J.E., Gallego R., Tovar S., Vazquez M.J., Garces M.F., Lopez M., Garcia-Caballero T., Tena-Sempere M., Nogueiras R. (2010). Adiponectin receptor 2 is regulated by nutritional status, leptin and pregnancy in a tissue-specific manner. Physiol. Behav..

[77] Perry B., Wang Y. (2012). Appetite regulation and weight control: The role of gut hormones. Nutr. Diabetes.

[78] Chaudhri O., Small C., Bloom S. (2006). Gastrointestinal hormones regulating appetite. Philos. Trans. R. Soc. Lond. B Biol. Sci..

[79] Maljaars P.W.J., Peters H.P.F., Mela D.J., NMasclee A.A.M. (2008). Ileal brake: A sensible food target for appetite control. A review. Physiol. Behav..

[80] Van Citters G.W., Lin H.C. (2006). Ileal brake: Neuropeptidergic control of intestinal transit. Curr. Gastroenterol. Rep..

[81] Khoshoo V., Brown S. (2002). Gastric emptying of two whey-based formulas of different energy density and its clinical implication in children with volume intolerance. Eur. J. Clin. Nutr..

[82] Stanstrup J., Schou S.S., Holmer-Jensen J., Hermansen K., Dragsted L.O. (2014). Whey protein delays gastric emptying and suppresses plasma fatty acids and their metabolites compared to casein, gluten, and fish protein. J. Proteome Res..

[83] Hale T.W., Hartmann P.E. (2007). Hale and Hartmann’s Textbook of Human Lactation.

[84] Van Avesaat M., Troost F.J., Ripken D., Hendriks H.F., Masclee A.A.M. (2015). Ileal brake activation: Macronutrient-specific effects on eating behavior?. Int. J. Obes..

[85] Boirie Y., Dangin M., Gachon P., Vasson M.P., Maubois J.L., Beaufrere B. (1997). Slow and fast dietary proteins differently modulate postprandial protein accretion. Proc. Natl. Acad. Sci. USA.

[86] Daniel H., Vohwinkel M., Rehner G. (1990). Effect of casein and beta-casomorphins on gastrointestinal motility in rats. J. Nutr..

[87] Stromqvist M., Falk F., Bergstrom S., Hansson L., Lonnerdal B., Normark S., Hernell O. (1995). Human milk kappa-casein and inhibition of helicobacter pylori adhesion to human gastric mucosa. J. Pediatr. Gastroenterol. Nutr..

[88] Francois F., Roper J., Joseph N., Pei Z., Chhada A., Shal J.R., Olivares De Perez A.Z., Perez-Perez G.I., Blaser M.J. (2011). The effect of *H. pylori* eradication on meal-associated changes in plasma ghrelin and leptin. BMC Gastroenterol..

[89] Montagne P., Cuilliere M.L., Mole C., Bene M.C., Faure G. (2001). Changes in lactoferrin and lysozyme levels in human milk during the first twelve weeks of lactation. Adv. Exp. Med. Biol..

[90] Rubio C.A. (2014). The natural antimicrobial enzyme lysozyme is up-regulated in gastrointestinal inflammatory conditions. Pathogens.

[91] Bol’shakova A.M., Shcherbakova E.G., Ivanova S.D., Medvedeva M.M., Zhuravleva T.P. (1984). Lysozyme in the feeding of premature infants with mixed pathology. Antibiotiki.

[92] Perrella S.L., Hepworth A.R., Simmer K.N., Hartmann P.E., Geddes D.T. (2014). Repeatability of gastric volume measurements and intragastric content using ultrasound in preterm infants. J. Pediatr. Gastroenterol. Nutr..

[93] Hellstrom P., Naslund E. (2001). Interactions between gastric emptying and satiety, with special reference to glucagon-like peptide-1. Physiol. Behav..

[94] Lorenz D.N. (1985). Gastric emptying of milk in rat pups. Am. J. Physiol..

[95] Kent J., Hepworth A., Sherriff J., Cox D., Mitoulas L., Hartmann P. (2013). Longitudinal changes in breastfeeding patterns from 1 to 6 months of lactation. Breastfeed. Med..

[96] Sepple C.P., Read N.W. (1989). Gastrointestinal correlates of the development of hunger in man. Appetite.

[97] Lawrence R.A., Lawrence R.M., Lawrence R.A., Lawrence R.M. (2011). Practical management of the mother-infant nursing couple. Breastfeeding: A Guide for Medical Profession.

[98] Natalucci G., Riedl S., Zidel T., Frisch H. (2005). Spontaneous 24-h ghrelin secretion pattern in fasting subjects: Maintenance of a meal-related pattern. Eur. J. Endocrinol..

[99] Degen L.P., Phillips S.F. (1996). Variability of gastrointestinal transit in healthy women and men. Gut.

[100] Butte N., Garza C., Smith E., Nichols B. (1984). Human milk intake and growth in exclusively breast-fed infants. J. Pediatr..

